# Iron, Oxidative Stress and Gestational Diabetes

**DOI:** 10.3390/nu6093968

**Published:** 2014-09-25

**Authors:** Taifeng Zhuang, Huijun Han, Zhenyu Yang

**Affiliations:** 1 Department of Neonatal Intensive Care Unit (NICU), Beijing Obstetrics and Gynecology Hospital, Capital Medical University, Beijing 100026, China; E-Mail: ztf19731972@126.com; 2 Department of Epidemiology and Biostatistics, Institute of Basic Medical Sciences Chinese Academy of Medical Sciences, School of Basic Medicine Peking Union Medical College, Beijing 100005, China; E-Mail: only_hhj@126.com; 3 Key Laboratory of Trace Element Nutrition of the Ministry of Health, National Institute of Nutrition and Food Safety, Chinese Center for Disease Control and Prevention, No. 27 Nanwei Road, Xicheng District, Beijing 100050, China

**Keywords:** iron, oxidative stress, lipid peroxidation, DNA damage, gestational diabetes

## Abstract

Both iron deficiency and hyperglycemia are highly prevalent globally for pregnant women. Iron supplementation is recommended during pregnancy to control iron deficiency. The purposes of the review are to assess the oxidative effects of iron supplementation and the potential relationship between iron nutrition and gestational diabetes. High doses of iron (~relative to 60 mg or more daily for adult humans) can induce lipid peroxidation *in vitro* and in animal studies. Pharmaceutical doses of iron supplements (e.g., 10× RDA or more for oral supplements or direct iron supplementation via injection or addition to the cell culture medium) for a short or long duration will induce DNA damage. Higher heme-iron intake or iron status measured by various biomarkers, especially serum ferritin, might contribute to greater risk of gestational diabetes, which may be mediated by iron oxidative stress though lipid oxidation and/or DNA damage. However, information is lacking about the effect of low dose iron supplementation (≤60 mg daily) on lipid peroxidation, DNA damage and gestational diabetes. Randomized trials of low-dose iron supplementation (≤60 mg daily) for pregnant women are warranted to test the relationship between iron oxidative stress and insulin resistance/gestational diabetes, especially for iron-replete women.

## 1. Introduction

Iron deficiency remains highly prevalent globally, especially for pre-school children and pregnant women. It was estimated that 19.2% of pregnant women had iron deficiency anemia worldwide [1]. Meta-analysis results showed that daily prenatal iron supplementation could increase maternal hemoglobin level and birth weight, which could lead to reducing the risk of maternal anemia and low birth weight [2,3]. Based on this evidence, the World Health Organization (WHO) recommends that all pregnant women should be given an iron supplement (30–60 mg elemental iron) daily throughout pregnancy in all settings [4]. Hyperglycemia is commonly seen during pregnancy and could be associated with various complications for both mothers and newborns. A recent study estimated that about 16.9% of pregnant women had hyperglycemia globally [5]. Iron overload has been considered a potential risk factor for type II diabetes [6]. The purposes of the review are to assess the oxidative effects of iron supplementation and the potential relationship between iron intake and gestational diabetes.

## 2. Iron and Oxidative Stress

As a transition metal, iron has five oxidation states (Fe^2+^–Fe^6+^) in addition to the ground state, and the most common states are Fe^2+^ and Fe^3+^ [7]. The unpaired electrons from iron make one-electron redox reactions possible. In the late 19th century, Fenton discovered that ferrous iron and hydrogen peroxide catalyze the oxidation of tartaric acid [8,9,10]. The work of Haber and Weiss subsequently showed that the hydroxyl free radical was produced by ferrous iron and hydrogen peroxide in a chain reaction, as shown below [9,11]. Reaction (1) is usually called the Fenton reaction, and the whole set of reactions is named the Haber–Weiss reactions. As the product of SOD-catalyzed reactions and non-enzyme reactions, hydrogen peroxide has been found in mitochondria, microsomes and chloroplasts from bacteria and has also been detected in the lens of the eye and exhaled air in humans [12]. The OH radical is highly reactive, and its reactivity is based on diffusion speed, because of its short life [13].

Fe^2+^ + H_2_O_2_ → Fe^3+^ + OH**^.^** + OH^−^ (1)

OH**^.^** + Fe^2+^ → Fe^3+^ + OH^−^ (2)

OH**^.^** + RH → H_2_O + R**^.^** (3)

OH**^.^** + LH → LOOH (4)

### 2.1. Iron and Lipid Peroxidation

The OH radical can non-specifically oxidize lipid molecules in the cell membrane and lipoprotein lipids (e.g., unsaturated fatty acids in phospholipids and cholesterol) to form LOOH in Reaction (4) mentioned above. Further, Fe^2+^ can catalyze the single-electron reduction of LOOH, the non-radical lipid hydroperoxide, to produce the LO (OLOO) free radical via one-electron oxidation [14,15]. Lipid peroxidation is initiated at this step. Some studies showed that ferric iron seems necessary to initiate lipid peroxidation [13,16] or some Fe^4+^ or Fe^5+^ complexes could be formed to initiate lipid peroxidation [17].

Either ferrous or ferric iron can increase lipid oxidation measured by malondialdehyde (MDA), thiobarbituric acid reactive substances (TBARS) or conjugated dienes in liposome model systems or human milk. In these studies, the concentration of iron ions ranged from 12 μM to 200 μM in the model systems or 1.8 mM added into human milk [13,18,19,20,21]. Ferritin iron has also been associated with elevated TBARS in the microsome system [22]. Antioxidants (e.g., ellagic acid, vitamin C, vitamin E, zolpidem or BHT) have been used to inhibit lipid oxidation induced by 10–1000 μM ferrous sulfate in the rat pheochromocytoma cell line, PC12, Caco-2 cells, murine corneal endothelial cells or liver homogenates [23,24,25,26]. Various iron compounds (iron gluconate, iron sucrose and iron dextran) are intravenously used to treat anemia due to poor iron absorption. One study found that each of these three iron compounds differed in initiating lipid oxidation in renal cortical tissue homogenate and was less reactive than ferric chloride [27]. Lipid oxidation induced by iron is dose-dependent within the range tested, varies among different iron compounds and is highest for ferric chloride [23,24,25,28].

Giving ferric nitrilotriacetate (Fe-NTA) intraperitoneally (9 mg Fe/kg body weight) can significantly increase liver and kidney MDA by 1.5–3-fold in the rat or mouse, and various antioxidants (e.g., garlic oil, pomegranate flower extractants, curcumin and farnesol) can attenuate this oxidative effect [29,30,31,32]. An acute iron overload model has been developed by injecting iron-dextran (300–500 mg /kg body weight) or ferrous sulfate (30 mg Fe/kg body weight) intraperitoneally in rats or rabbits. This technique has shown that TBARS levels significantly increase in liver, kidney and plasma [33,34,35,36,37,38,39,40,41]. 

A chronic iron overload rat model has been developed that involves oral iron supplementation with carbonyl iron (2.5% w/w) for six weeks. In this model, the level of TBARS is increased ~15-fold in liver, but not in plasma [42]. Iron supplements (3 g/kg or 30 g/kg Fe as pentacarbonyl iron in the diet) given to rats with dextran sulfate sodium (DSS)-induced colitis for one week caused an increase in lipid peroxide concentrations in both plasma and colon (measured by free MDA and 4-hydroxyalkenals), even at the lower iron concentration (3 g/kg) [43]. Compared to non-supplemented rats, iron supplementation (8 mg Fe/day as ferrous sulfate for 21 days) significantly increased lipid oxidation measured by breath ethane level, but not MDA, in both normal and iron deficient rats [44]. The same study also showed that iron deficiency elevated lipid peroxidation measured by both breath ethane and liver and kidney MDA.

The association between iron supplementation and acute phase lipid peroxidation is not conclusive in humans. In one study, giving one dose of iron (45 mg Fe as ferrous sulfate) caused no significant increase in lipid peroxidation measured by plasma isoprostane concentration. Another iron supplementation study also did not find changes in concentrations of isoprostane or TBARS on days 1, 2 and 7 after giving a series of doses of iron increasing from 0 mg to 240 mg every week, with 1–2 weeks washout in between each dose [45].

A randomized controlled trial was conducted to assess the effect of iron supplementation during the third trimester of pregnancy on iron status and lipid peroxidation [46]. Subjects (*n* = 54) were randomly assigned to the: (1) supplementation group (100 mg Fe as ferrous fumarate and 500 mg vitamin C as ascorbate daily for the last trimester); or (2) control group (without any treatment). Baseline iron status information was not available. Serum iron concentration and hemoglobin concentration, but not serum ferritin concentration, were significantly higher in the supplementation group than in the control group. No subjects had anemia or low iron stores at delivery. Lipid peroxidation, measured by TBARS, was significantly greater in the supplementation group than in the control group. Plasma α-tocopherol concentration decreased significantly in the supplementation group compared to the control group. However, an effect of vitamin C on lipid peroxidation cannot be ruled out in this study, because vitamin C not only enhances iron absorption, but can also increase intracellular peroxidation via an iron-dependent reaction [47]. As an anti-oxidant, vitamin C can also affect lipid oxidation dose-dependently [48].

The effect of an iron chelator (desferrioxamine) on lipid peroxidation was assessed in a randomized clinical trial [49]. Cardiopulmonary bypass patients (*n* = 20) were randomly assigned to the: (1) desferrioxamine treatment group during cardiopulmonary bypass surgery; or (2) control group. Desferrioxamine treatment significantly decreased LDL-TBARS.

Taken together, these studies support the hypothesis that high doses of iron (~relative to 60 mg or more daily for adult humans) might induce lipid peroxidation *in vitro* and in animal studies. However, few data showed lipid peroxidation of lower doses of iron (≤60 mg daily), especially in humans. 

### 2.2. Iron and DNA Damage

The majority of hydrogen peroxide-mediated DNA damage could be due to on-site hydroxyl radicals, which are catalyzed by iron associated with DNA [7,50,51,52]. Hydroxyl radicals can attack both DNA bases (pyrimidines and purines) and sugars (reviewed in [53]). A large amount of end products of DNA damage, found in *in vitro* studies, are formed during oxidative reactions and subsequent reactions [53,54,55]. The most commonly studied biomarkers of DNA oxidative products or DNA damage are 7,8-dihydro-8-oxo-2-deoxyguanosine (8-OH-dG) and its related base (8-oxo-guanine).

Fe^2+^/H_2_O_2_ (Fenton reactant) has been used to induce intracellular oxidative damage and to build DNA damage cell models in numerous studies. When 100–250 μM iron alone were added into cell culture medium to treat T-cells, cerebellar granule cells or liver mitochondria, DNA damage, which was determined by 8-OHdG concentration, comet assay or DNA ladder, was significantly greater than in controls [56,57,58].

Iron chelators have also been used to investigate the potential role of iron in DNA oxidative damage induced by hydrogen peroxide in cell models. 1,10-phenanthroline is a chelator that can bind both ferrous iron and cuprous copper, and 2,9-dimethyl-l,10-phenanthroline (neocuproine) is another chelator with a structure similar to 1,10-phenanthroline, except for two more methyl groups, which can only bind copper. When both chelators were used to inhibit DNA damage induced by the Fenton reaction, only 1,10-phenanthroline could protect DNA strand breaks from hydrogen peroxide in human fibroblast cells, which indicates that iron may be the main catalyst of the Fenton reaction to induce intracellular DNA damage [59]. A similar study showed that intracellular chelators (either desferrioxamine (DFO) or 1,10-phenanthroline) can inhibit DNA damage induced by hydrogen peroxide in Jurkat T-cells, but this is not true for extracellular iron chelators, such as diethylenetriaminepentaacetic acid (DTPA).

In animal models, investigators have also found that there are positive associations between DNA damage and iron treatment, including one dose of injected iron, short-term oral iron supplementation (15 days and 34 days) and long-term dietary iron load (12 months and 20 months). At 24 h after intracerebral injection of iron (16.8 μg Fe as ferrous chloride) to rats, DNA damage in brain basal ganglia was obvious by measuring stained DNA polymerase I-mediated biotin-dATP nick translation [60]. After one dose of an oral 8-mg iron supplement, mucosal cell DNA damage was detected using DNA ladder in a rat model [61]. It has also been shown in rats that an 8-mg iron supplementation for 15 days or 34 days significantly increases DNA damage, measured by lower mucosal radioactive labeled thymidine incorporation [61] or liver mitochondria DNA fragments [62]. It is interesting that iron deficiency significantly elevates DNA damage in liver mitochondria, but not gastrointestinal mucosal cells, compared to rats with normal iron status [61,62].

Oral iron supplementation (300 mg Fe/kg diet given as ferrous sulfate for 12 months) significantly increased the concentration of liver modified DNA bases (dihydrothymine, 5-OH uracil, 5-OH methyluracil and fapyadenine) in C3H mice, compared to those fed a 100-mg Fe/kg diet [63]. 

In another study, rats were randomly assigned to a control group or an iron overload group (2% Fe as carbonyl iron during the first eight months, then 2.5% Fe between eight and 12 months, then 0.5% Fe as dicyclopentadienyl ferrocene for the rest of the study to ensure hepatic iron overload). The whole study lasted for 32 months. At 20 months, the 8-OHdG concentration and rate of DNA unwinding were significantly greater in the iron overload group than in the control group [64].

Forty healthy adults were given iron supplements (12.5 mg Fe as ferrous glycine sulfate) daily for six weeks. Iron status (ferritin, transferrin bound iron (TBI) and transferrin saturation) and DNA damage (8-OHG, 8-OHA, 5-OHC, FAPy G, FAPy A, 5OHU, thymine glycol) were not different pre- and post-supplementation. However, there was a significant correlation between TBI and modified DNA base concentration before the study (*r* = 0.48, *p* = 0.002) [65]. Recent studies showed a similar positive association between body iron stores and DNA damage (8-OHdG), even within the normal range of serum ferritin [66,67].

Overall, these studies suggest that pharmaceutical doses of iron supplements (e.g., 10× RDA or more for oral supplements, or direct iron supplementation via injection or addition to the cell culture medium) for a short or long duration will induce DNA damage, measured by different techniques. However, information is lacking about the effect of low dose iron supplementation (1× RDA–10× RDA) on DNA damage. This is the dose range of iron supplements usually used to prevent or treat iron deficiency in humans. More focus should be given to this topic. Furthermore, the relationship between iron deficiency and DNA damage should be explored in human studies.

## 3. Iron and Gestational Diabetes 

A systematic search of published literature from January 1, 1966, through August 25, 2014, was conducted, using the U.S. national Library of medicine’s MEDLINE/PubMed bibliographic search engine (http://www.pubmed.gov). Search terms were the combinations of (“iron” (MeSH (Medical Subject Heading) Terms) or “iron” (TIAB(Title/Abstract)) and (“diabetes, gestational” (MeSH Terms) or (“gestational” (TIAB) and “diabetes” (TIAB)). Only human studies were selected. The searches yielded 85 publications, of which 32 citations were reviewed, and the remaining were excluded because they were not relative to the topic.

The pancreatic beta-cell is susceptible to oxidative stress. A recent review showed that iron-related oxidative stress might contribute to gestational diabetes [68]. Lipid oxidation and DNA damage might be the intermediate pathways of the relationship between iron nutrition and gestational diabetes. A cross-sectional study found that MDA concentration was higher and SOD activity was lower in gestational diabetes patients than normal pregnant women. A nested case-control study assessed the relationship between urinary 8-OHdG concentration and the risk of gestational diabetes. The highest quartiles of the 8-OHdG group had 3.8-fold higher adjusted odds of gestational diabetes than the lowest quartiles. Non-transferrin bound iron (NTBI) was positively associated with adipocyte insulin resistance in a cross-sectional study.

Dietary heme iron intake, but not non-heme iron intake, and supplemental iron intake increased the risk of gestational diabetes in a few studies [69,70]. Every 1 mg of heme iron intake increased the adjusted odds of gestational diabetes by 51% [71]. Another study showed that high total iron intake (mean total dietary iron intake of 136.2 mg per day) increased the adjusted odds of gestational diabetes by 2.35-times for non-anemic women, compared with those with low iron intake (mean total dietary iron intake of 36.0 mg or 11.6 mg per day) [72]. Findings regarding the relationship between iron supplement and gestational diabetes have varied in other studies. An observational study found that iron supplementation was associated with impaired glucose metabolism [73]. A randomized controlled trial in Hong Kong found that iron supplementation (60 mg per day) from early pregnancy did not increase the risk of gestational diabetes at 28 weeks of gestation (OR: 1.04 (0.7, 1.53)) [74]. In the study, subjects had hemoglobin levels between 8 g/dL and 14 g/dL, which could differentiate the effects of iron supplementation on iron-deficient subjects and iron-replete subjects. A large-scale randomized controlled trial in Finland was published very recently on the effects of iron supplementation on gestational diabetes [75]. The results showed that routine iron supplementation (100 mg daily throughout pregnancy regardless of anemia status) did not increase the risk of glucose intolerance-related outcomes. In that study, the control group was given iron supplements (50 mg twice daily) if a pregnant woman was diagnosed as anemic; the maximum duration of iron supplementation was two months, and less than 20% of the subjects used iron supplements during the pregnancy. At 36 weeks of gestation, the hemoglobin level was significantly greater in the routine iron supplementation group than in the control group (131 g/L *vs.* 125 g/L). As stated by those authors, one of the main limitations is that the iron status biomarker was not measured in the trial. Both randomized controlled trials did not support that iron supplementation might increase the risk of gestational diabetes. However, it is unknown whether the iron status of the subjects modified the relationship.

The reason remains unclear why heme iron intake, and not other types of iron intake, including supplemental iron, was positively associated with a higher risk of gestational diabetes. Is this due to confounders from observational studies, as randomized controlled trials did not show the association? One study showed that gestational diabetes subjects had higher iron intake than the control subjects [76]. Prospective cohort study results did not support the reverse causation. Or does heme iron differ from other source of iron in the relationship? As heme iron is more bioavailable than non-heme iron, the body iron status increased more efficiently than other types of iron. Or might heme, not iron per se, be related to glucose metabolism impairment? Further studies could test the hypotheses of iron intake, either heme or non-heme iron, on iron status, oxidative stress and gestational diabetes.

Various biomarkers (serum iron, ferritin, hepcidin) were used to assess the relationship between iron status and gestational diabetes. A higher serum iron concentration was related to a higher risk of gestational diabetes [77,78]. Another cross-sectional study found that the gestational diabetes group had a lower serum iron concentration than the control group [79] or no association was found [80,81]. Increased plasma ferritin, an iron storage marker, has been positively associated with an impaired oral glucose tolerance test in a few studies [77,82,83,84,85,86]. Another study found that the ferritin level at delivery was positively related to the risk of gestational diabetes, but the plasma ferritin level was not associated with the transferrin receptor level [84]. A prospective cohort study also found that serum ferritin concentration was significantly greater in gestational diabetes subjects than those without GDM. However the relationship was attenuated after adjusting pre-pregnancy BMI [83]. The serum hepcidin level was elevated in gestational diabetic women [87]. Hepcidin is a small peptide hormone responsible for iron absorption regulation. A case-control study showed that gestational diabetes was inversely related to iron deficiency anemia [88]. A higher hemoglobin concentration at the first prenatal visit was associated with greater risk of gestational diabetes in a prospective cohort study [89]. Gene polymorphism (C282Y) of hereditary hemochromatosis (excess iron accumulation) or other iron overload hemoglobinopathies (e.g., thalassemia, sickle cell trait) were associated with GDM [90,91]. However, another case-control study did not find a relationship between iron status (assessed by using serum iron, ferritin and total iron binding capacity) and glucose intolerance [92]. Overall, serum ferritin might be positively associated with the risk of gestational diabetes, although the association between other iron status biomarkers (e.g., serum iron) and the risk of gestational diabetes was not consistent. One reason could be due to ferritin possibly being a better indicator for assessing the relationship, as ferritin reflects body iron storage and might be associated with lipid oxidation and DNA damage. In addition, ferritin is an acute phase protein, which may be related to inflammation and may have biased the relationship between iron status and gestational diabetes. Residual confounders might have the potential to affect the conclusions on these observational studies.

## 4. Conclusions 

In summary, it remains unclear whether lipid oxidation and DNA damage due to excess iron in the body or high-dose iron intake might be associated with the development of gestational diabetes. Low dose iron supplements (≤60 mg daily) during pregnancy for subjects with adequate iron status warrants further studies. Randomized trials of low dose iron supplementation (≤60 mg daily) for pregnant women could test the relationship between iron oxidative stress and insulin resistance and gestational diabetes, especially for iron-replete women.
